# Self-perceived sleepiness in emergency training physicians: prevalence and relationship with quality of life

**DOI:** 10.1186/1745-6673-8-24

**Published:** 2013-09-21

**Authors:** Jihane Belayachi, Oumama Benjelloun, Naoufel Madani, Khalid Abidi, Tarek Dendane, Amine Ali Zeggwagh, Redouane Abouqal

**Affiliations:** 1 Medical Emergency Department, Ibn Sina University Hospital, 10000 Rabat, Morocco; 2 Medical Intensive Care Unit, Ibn Sina University Hospital, 10000 Rabat, Morocco; 3 Faculty of Medicine, Laboratory of Biostatistics Clinical and Epidemiological Research, University Mohamed V Souissi, 10000 Rabat, Morocco

**Keywords:** Emergency, Quality of life, Sleepiness, Training physicians

## Abstract

**Introduction:**

Sleep deprivation among training physicians is of growing concern; training physicians are susceptible due to their prolonged work hours and rotating work schedules. The aim of this study was to determine the prevalence of self-perceived sleepiness in emergency training physicians, and to establish a relationship between self-perceived sleepiness, and quality of life.

**Methods:**

Prospective survey in Ibn Sina University hospital Center in Morocco from January to April 2011 was conducted. Questionnaires pertaining to socio-demographic, general, and sleep characteristics were completed by training physician who ensured emergency service during the month preceding the survey. They completed the Epworth sleepiness scale (ESS) which assessed the self-perceived sleepiness, and the EuroQol-5 dimensions (EQ-5D) scale which assessed the general quality of life.

**Results:**

Total 81 subjects (49 men and 32 women) were enrolled with mean age of 26.1 ± 3.4 years. No sleepiness was found in 24.7% (n = 20), excessive sleepiness 39.5% (n = 32), and severe sleepiness in 35.8% (n = 29) of training physicians. After adjusting for multiple confounding variables, four independent variables were associated with poorer quality of life index in training physician; unmarried (ß −0.2, 95% CI −0.36 to −0.02; *P* = 0.02), no physic exercise (ß −0.2, 95% CI −0.39 to 0.006; *P* = 0.04), shift-off sleep hour less than 6 hours (ß −0.13, 95% CI −0.24 to −0.02; *P* = 0.01), and severe sleep deprivation(ß −0.2, 95% CI −0.38 to −0.2; *P* = 0.02).

**Conclusion:**

Nearly two third of training physicians had suffered from sleepiness. There is an association between poor quality of life and severe sleepiness in unmarried physicians, sleeping less than 6 hours in shift-off day, and doing no physical activity.

## Introduction

Training physicians are susceptible to fatigue and sleep deprivation due to their prolonged work hours and rotating work schedules [1]. Residency work hours have become the focus of numerous comprehensive reviews [2-8]. The effect of sleep loss in the context of medical training is a topic that has generated considerable interest, as well as controversy, over the past two decades. Research on physicians sleep deprivation has primarily focused on the detrimental impact of fatigue on patient care [9,10]. Various specialties, including Internal Medicine [6,7], Obstetrics and Gynecology [11], Pediatrics [12], have shown sleep deprivation among residents negatively affects performance [13,14]. Studies have identified many domains affected by fatigue impacting both physical and psychological disorders, ranging from an increased risk of depression [15], and heart disease [16], to professionalism, learning, errors, and completion of complex cognitive tasks [17-19]. The increased incidence of automobile accidents among interns and residents is also well documented [20]. To our knowledge, no publications concerning the relationship between self-perceived sleepiness and quality of life of emergency training physicians were available, and no one concerning sleepiness in Moroccan physicians.

Therefore, the purpose of the present study was to determine the prevalence of self-perceived sleepiness using the Arabic version of Epworth sleepiness scale (ESS) in emergency training physicians, and to establish a relationship between self-perceived sleepiness, and quality of life using the valid Arabic version of EuroQol-5dimensions (EQ-5D).

## Methods

### Study design and participants

From January to April 2011, we conducted a prospective survey in Ibn Sina University hospital Center (CHIS) in Morocco.

Training physicians who realized at least one overnight care per week during the month before the survey were included in the study. Training physicians who realized less than one overnight care per week were excluded.

According to practice standard in our institute, training physician rotate between medical and surgery departments every six months, and ensured emergency service regularly (3 to 4 times monthly). Emergency service consisted to 12 to 24 hours night shift. The shift are required in emergency department during the two firsts years of training, beyond they become optional.

### Setting

Ibn Sina Hospital Center (CHIS) is the referral for habitants in Western-north Morocco; it is a tertiary – stage hospital. It is a structure consisting of 10 hospitals. CHIS are the largest hospital facility in Morocco: 2,489 beds, 6,483 health professionals, 401,994 consultations per year, 28 432 operations per year, 23 697 deliveries per year. The study was approved by the Rabat University ethics committees Participation of the doctors was voluntary, and written consent was given.

### Data collection

Because of the potential for respondents to provide incriminating responses, we elected to conduct the survey with anonymous responses. The participants filled out a questionnaire, and completed information concerning socio-demographic, general, and sleep characteristics. Socio-demographic data included age (per years), sex (male, female), marital status (married, unmarried), and if had children (yes, no). General characteristics included training physicians year (first year or second year and more), work hour (per day); caffeine intake (yes, no); tabac (yes, no); ethylism (yes, no), exercise (yes, no); change in body weight (yes, no); and if yes weight gain or weight loss (per Kg). Sleep characteristics (shift-off, and shift-on sleep hours (≤4 h, 4-6 h, >6 h), a self-perceived sleepiness.

### Instruments

Training physicians completed two instruments: the ESS which assessed the self-perceived sleepiness and the EQ-5D scale which assessed the general quality of life based on experiences over the previous month.

### The epworth sleepiness scale (ESS)

The ESS is a standardized validated questionnaire that assesses the likelihood that the subject will fall asleep during certain activities [21]. It presents the respondent with eight situations requesting selection from a four-point Likert scale. The response to each question is obtained on a scale from 0 (not at all likely to fall asleep) to 3 (very likely to fall asleep), the resulting total score is between 0 and 24. The ESS ranges from 0 to 24, and can be used to categorize respondents as normal (ESS ≤ 10), suffering from excessive sleepiness (ESS 11–16), or suffering from severe sleepiness (ESS > 16). The ESS has good internal and test retest reliability and has also shown moderate correlation with objective sleep-propensity tests [22,23].

The ESS is a simple, self-administered questionnaire which is shown to provide a measurement of the subject’s general level of daytime sleepiness.

Because of no available Arabic version of the ESS in the beginning of the study translation procedure followed by a transcultural adaptation were taken following international guidelines [24]. These following steps were used, in the first phase; the ESS was translated by three bilingual (native Arabic, and English) individuals. Once the three translations were completed, one unified translation of the ESS was created by a committee consisting of the translators and three further individuals not involved in the translation process (a sociologist and two epidemiologists). Then, the Arabic version of the ESS was back translated by a native English speaker living in morocco, who was unaware of the original English document. Once the back translation completed, the committee reconvened to review and resolve the discrepancies between back translation and the original document. Finally, a pretest was conducted with a group of lay native Arabic speakers (30 subjects). Discrepancies were resolved by group consensus. Globally, the adaptation did not cause any particular problems. The internal consistency levels (chronbach’s alpha coefficients) of arabic version of the ESS was 0.72.

### EQ 5D scale

The first part of the EQ-5D contains a description of the health state in 5 dimensions or items: Mobility, Self-care, Usual activities, Pain/discomfort, and Anxiety/Depression levels of severity for each item: 1 (No problems), 2 (Some problems) and 3 (Unable to do/ Extreme problems). For each item, the respondent must indicate the level of severity that best describes his/her personal health state at the time of giving the answers. The subject’s global health state is finally defined as the combination of the level of problems described for each of the five dimensions contained in the EQ-5D. Therefore, it classifies a respondent’s health status into one of 243 (35) health states. Each health state can be assigned a weighted utility score based on different scoring systems. The unweighted scoring rule based solely on answers provided by subjects to the descriptive system. The values are ranging from −0.59 (the lowest level on each dimension) to 1 (The highest level on each dimension). Negative values indicate that some health states are valued worse than death [25].

The second part of the EQ-5D questionnaire is a visual analogue scale (EQ-VAS) [26].The EQ-VAS is a vertical, graduated (0–100 points) 20 cm ‘thermometer’, with 100 representing ‘best imaginable health state’ and 0 representing ‘worst imaginable health state’. The respondent rated his health on the day of the survey using both the self-classifier system and the VAS.

The Arabic version of the EQ-5D for Morocco was adapted from the United Arab Emirates Arabic version using EuroQol group guidelines and input [27]. The Arabic version for Morocco of the EQ-5D is reliable and valid [28].

### Statistical analysis

Continuous variables are presented as mean ± standard deviation for variables with a normal distribution, and as median and interquartile range (IQR) for variables with skewed distributions. The normality of the distribution was tested by the Kolmogorov-Smirnov test with Lilliefors correction. For categorical variables, the percentages of patients in each category were calculated. The internal consistency of the ESS items was assessed using Chronbach’s coefficient alpha; a high alpha coefficient (≥0.70) suggests that the items within a scale measure the same construct and support the construct validity [29,30]. Concerning univariate analysis, for statistical differences between the three subgroups of ESS (normal sleep, excessive sleepiness, and severe sleepiness), the One Way Analysis Of Variance (ANOVA), and Chi Square test were performed. Univariate linear regression was also used to identify the relationship between EQ-5D and socio-demographic general, and sleep characteristics variables. Variables with P values ≤ 0.20 in the univariate analysis were tested in the multivariate analysis. Multivariate analysis was performed using forward multiple linear regression analysis. A two-tailed *P* value < 0.05 was considered statistically significant. Statistical analyses were carried out using SPSS for Windows (SPSS, Inc., Chicago, IL, USA).

## Results

### Characteristics of subjects

Of the 120 questionnaires distributed, 95 (79.2%) were returned. twenty five questionnaires were rejected (20.8%) for following reasons: chronic medical illnesses (n = 3), and incompleteness (n = 11). Then, 81 remaining participants were enrolled. Table 1 summarizes the sociodemographics, general, sleep characteristics of the total study population as well as the “no sleepiness”, “severe sleepiness”, and “excessive sleepiness” groups.

**Table 1:** The summary of socio demographics, general, sleep characteristics of the total study population as well as the “no sleepiness”, “severe sleepiness”, and “excessive sleepiness” groups

**Variables**	**n (%), mean ± SD**	**No sleepiness n = 20**	**Excessive sleepiness n = 32**	**Severe sleepiness n = 29**	***P***
**Sociodemographics characteristics**					
**Age** (per years)	26.1 ± 3.4	26.9 ± 4	26.2 ± 3.5	26.1 ± 3.2	0.33
**Sex**					0.04
Male	49(60.5)	16(80)	20(62.5)	13(44.8)	
Female	32(39.5)	4(20)	12(37.5)	16(55.2)	
**Marital status**					0.92
Married	14(17.3)	3(15)	6(18.8)	5(17.2)	
Unmarried	67(82.7)	17(85)	26(81.3)	24(82.8)	
**Had children**					
Yes	4(21.8)	---	---	---	
**General characteristics**					
**Training year**					0.01
First year	58	10(17.2)	22(38)	26(44.8)	
Second year and more	22	9(41)	10(45.5)	3(13.6)	
**Work hours** (per day)	8.5 ± 2.3	8.7 ± 2.3	8.1 ± 2.3	8.6 ± 2.3	0.61
**Tabac**					0.09
Yes	7(8.6)	2(10)	5(15.6)	0	
No	74(91.4)	18(90)	27(84.4)	29(100)	
**Caffeine intake**					0.43
Yes	40(49.4)	12(60)	16(50)	12(41.4)	
No	41(50.6)	8(40)	16(50)	17(58.6)	
**Ethylism**					0.21
Yes	1(1.2)	---	---	---	
No	80(98.8)	---	---	---	
**Exercice**					
Yes	12(15.2)	4(21.1)	2(6.5)	6(20.7)	0.22
No	67(84.8)	15(78.9)	29(93.5)	23(79.3)	
**Change in body weight**					0.02
Yes	61(76.3)	11(55)	22(78.6)	28(78.5)	
No	19(23.8)	9(45)	4(12.5)	6(21.4)	
Weight gain (per kg)	5.5 ± 3	5.6 ± 2.7	5.5 ± 3.3	5.4 ± 3.7	0.75
Weight loss (per kg)	5.6 ± 4	2.2 ± 0.9	6 ±4.9	6.1 ± 3.6	0.21
**Sleep characteristics**					
**Shift-off sleep hours**					0.17
≤4 h	1(1.3)	1(5)	0	0	
4–6 h	30(39)	4(20)	14(45.2)	12(46.2)	
>6 h	46(59.7)	15(75)	17(54.8)	14(53.8)	
**Shift-on sleep hours**					0.01
≤4 h	66(85.7)	14(70)	28(90.3)	24(92.3)	
4–6 h	4(5.2)	4(20)	0	0	
>6 h	7(9.1)	2(10)	3(9.7)	2(7.7)	
**General quality of life**					
EQ-5D Index	0.61 ± 0.3	0.7 ± 0.3	0.6 ± 0.3	0.5 ± 0.2	0.35
EQ-VAS	60 ± 20	65.7 ± 21.5	58.9 ± 20.3	58 ± 17.4	0.34

*n* frequence, *SD* standard deviation, *IQR* interquantil range, *%* pourcentage, *Kg* kilogramme.

*EQ5D* Euroqol 5 dimension, *VAS* visual analogic scale.

### Descriptive statistics of the subscales

#### Epworth sleepiness scale

The internal consistency levels (chronbach’s alpha coefficients) of arabic version of the ESS was 0.72. The questionnaire had been generally well accepted by all participants. The mean duration of administration was 1 minutes. It has been correctly completed by 81 of physicians with no missing or confusing item. The mean (± SD) value of the total score was 13.2 ±4.6**.** The highest mean value was observed for the item” Lying down to rest in the afternoon when circumstances permit”. No sleepiness was found in 23.4% (n = 19), excessive sleepiness 39.5% (n = 32), and severe sleepiness in 27.2% (n = 22) of training physicians.

#### Euro-Qol 5d

The questionnaire had been generally well accepted by all participants. The mean duration of administration was 2 min. it has been correctly completed by 98.8% (n = 80) of physicians with no missing or confusing item. The mean (±SD) value of the total EQ-5D Index score was 0.67 ± 0.24 and ranged from −0.11 to 1, and the median [IQR] value of the total EQ-VAS score was 70[60–80], and ranged from 4 to 100. Score distributions of the EQ-5D self-classifier in doctors were summarized in Table 2.

**Table 2:** Score distributions of the EQ-5D self-classifier in training physicians

**EQ-5D dimensions**	**Responses; n (%)**		
	**No problems**	**Moderate problems**	**Extreme problems**
Mobility	53(65.4)	25(30.9)	2(2.5)
Self-care	73(90.1)	8(9.9)	0
Usual activities	33(40.7)	39(48.1)	9(11.1)
Pain/discomfort	33(40.7)	47(58.0)	1(1.2)
Anxiety/depression	32(39.5)	40(49.4)	8(9.9)

*n* frequence, *% *pourcentage.

### Relationship between self-perceived sleep and quality of life

#### Univariate analysis

Concerning self-perceived sleepiness; Female gender of physicians was associated with severity of sleepiness (no sleepiness 4(20%), excessive sleepiness 12(37.5%), severe sleepiness 16(55.2%); *P* = 0,04). Concerning general characteristics; the severity of sleepiness was associated with: first year training physicians (no sleepiness 10(17.2%), excessive sleepiness 22(38%), severe sleepiness 26(44.8%); *P* = 0,01); change in body weight (no sleepiness 11(55%), excessive sleepiness 22(78.5%), severe sleepiness 28(78.5%); *P* = 0,02), and shift on sleep less than 4 hours (no sleepiness 14(70%), excessive sleepiness 28(90.3%), severe sleepiness 24 (92.3%); *P* = 0,02) . Table 1 summarizes the sociodemographics, general, and sleeps characteristics in all training physicians and depending on severity of sleepiness.

Concerning EQ-5D index, factors associated with poorer quality of life were; female gender of physicians (ß −0.12, 95% CI −0.25 to 0 ; *P* = 0.05), shift-off (ß −0.12, 95% CI −0.2 to −0.005 ; *P* = 0.04), and shift-on (ß −0.3, 95% CI −0.6 to −0.006 ; *P* = 0.04) sleep hour between 4 and 6 hours, and the absence of physical exercise (ß −0.18, 95% CI −0.009 to −0.35; *P* = 0.04).

Concerning EQ-5D VAS, in univariate analysis, factors associated with poorer quality of life were; shift-off sleep hour between 4 and 6 hours (ß −9.6, 95% CI −18.4 to −0.8; *P* = 0.03), shift-on sleep hour less than 4 hours (ß −14.6, 95% CI −29.7 to 0.3; *P* = 0.05), and no caffeine intake (ß −7.8, 95% CI −19.01 to −2.3; *P* = 0.01). Univariate analysis of socio-demographic, general and sleep characteristics affecting quality of life of participants was summarized in Table 3.

**Table 3:** Univariate analysis of socio-demographic, general and sleep caracteristics affecting quality of life of participants

**Variables**	**EQ index**			**EQ VAS**		
	**β**	**95% CI**	***P***	**β**	**95% CI**	***P***
**Socio-demographioc characteristics**						
**Age** (per year)	0.11	−0.01; 0.03	0.22	0.3	−0.9; 1.5	0.64
**Sex**						
Male	0	----	----	7.7	−0.9; 16.4	0.08
Female	−0.12	−0.25; 0	0.05	0	----	----
**Marital status**						
Married	0	----	----	0	----	----
Unmarried	−0.78	−0.24; 0.09	0.36	0.4	−10.9; 11.6	0.09
**General characteristics**						
**Training year**						
First year	−0.11	−0.24; 0.37	0.15	−5	−14; 4.5	0.30
Second year and more	0	---	---	0	----	----
**Work hours** (per day)	0	−0.002; 0	0.26	−0.01	−0.07; 0.04	0.55
**Tabac**						
Yes	0.08	−0.13; 0.3	0.45	4.3	−10.7; 19.5	0.5
No	0	---	----	0	----	----
**Caffeine intake**						
Yes	0	----	----	0	----	----
No	0.09	−0.2; 0.03	0.14	−10.6	−19.01; -2.3	0.01
**Exercice**						
Yes	0	----	----	0	----	----
No	−0.18	−0.009; -0.35	0.04	−7.8	−19.8; 4.1	0.19
**Change in body weight**						
Yes	−0.07	−0.2; 0.07	0.33	−2.05	−12.4; 8.3	0.7
No	0	----	----	0	---	----
**Weight gain (per kg)**	−0.01	−0.05; 0.01	0.36	−0.03	−3.05; 2.9	0.9
**Weight loss (per kg)**	−0.007	−0.03; 0.01	0,6	0.17	−1.5; 1.9	0.8
**Sleep characteristics**						
**Shift- off sleep hour**						
≤4 h	−0.31	−0.8; 0.2	0.23	−14.3	−52.2; 23.6	0.46
4-6 h	−0.12	−0.2; -0.005	0.04	−9.6	−18.4; -0.8	0.03
>6 h	0	----	----	0	----	----
**Shift- on sleep hour**						
≤4 h	−0.1	−0.3; 0.1	0.32	−14.6	−29.7; 0.3	0.05
4-6 h	−0.3	−0.6; -0.006	0.04	−13.5	−37.2; 10.1	0.26
>6 h	0	----	----	0	----	----
**Self perceived sleepiness**						
No sleepiness	0.1	−0.04; 0.31	0.14	10.5	−1.3; 22.3	0.08
Excessive sleepiness	0.0 3	−0.11; 0.19	0.63	7.08	−3.5; 17.6	0.18
Severe sleepiness	0	----	----	0	----	----

*SD* standard deviation, *h* hours, *EQ5D* Euroqol 5 dimension *EQ index* Euroqol index, *EQ VAS* Euroqolvisual analogic scale, *Kg* kilogramme, *CI* confidence interval. β: β coefficient from the multivariate lineal regression, after adjustment by all relevant variables. Positive values indicate better quality of life on that domain for that category; negative values indicate poorer quality of life compared with the reference category. 0 indicated reference.

### Multivariate analysis

Concerning EQ 5D index: After adjusting for multiple confounding variables, four independent variables were associated with poorer quality of life; single status (ß −0.2, 95% CI −0.36 to −0.02; *P* = 0.02), shift-off sleep hour less than 6 hours (ß −0.13, 95% CI −0.24 to −0.02; *P* = 0.01), no physic exercise (ß −0.2, 95% CI −0.39 to 0.006; *P* = 0.04), and severe sleep deprivation (ß −0.2, 95% CI −0.38 to −0.2 ; *P* = 0.02).

Concerning EQ 5D VAS: no variable affected quality of life in multivariate analysis. The result of forward multiple linear regression analysis concerning variables affecting quality of life (EQ-5D index) was summarized in Table 4.

**Table 4:** Multiple linear regression analysis adjusting for variables affecting quality of life (EQ-5D index)

**Variables**	**EQ - 5D index**		
	**β**	**95% CI**	***P***
**Marital status**			
Married	0	---	---
Unmarried	−0.2	−0.36; -0.02	0.02
**Exercice**			
Yes	0	---	
No	−0.2	−0.39; 0.006	0.04
**Sleep characteristics**			
**Shift- off sleep hour**			
≤4 h	−0.6	−1.06; -0.1	0.01
4-6 h	−0.13	−0.24; -0.02	0.02
>6 h	0	---	---
**Self perceived sleepiness**			
No sleepiness	0	---	---
Excessive sleepiness	0.06	−0.07; 0.2	0.3
Severe sleepiness	−0.2	−0.38; -0.2	0.02

*EQ-5D* Euroqol 5 dimension, *CI* confidence interval. β: β coefficient from the multivariate lineal regression, after adjustment by all relevant variables. Positive values indicate better quality of life on that domain for that category; negative values indicate poorer quality of life compared with the reference category. 0 indicated reference.

## Discussion

This study reports the results of one of very few studies who establish a relationship between self-perceived sleepiness, and general quality of life using the valid Arabic version of EQ-5D, and the first Moroccan study concerning self-perceived sleepiness in emergency physicians using the Arabic version of ESS.

Almost a third (66,7%) of overall participants had suffered from sleepiness. The current study demonstrates a clear association between poor quality of life and severe sleepiness in unmarried physicians, sleeping less than 6 hours in shift-off day, and doing no physical activity.

Sleep hours less than 6 hours in shift-off day was associated with poorer quality of life. In addition to restricted sleep, inadequate off-duty “recovery sleep” is a factor that potentially magnifies the effects of inadequate sleep in medical setting [31].

It is obvious that a healthy lifestyle improved the quality of life. In effect regular physical exercise is an important lifestyle factor that could enhance or maintain physical fitness and provide many health benefits [32]. Subject who exercised regularly with sufficient intensity were less likely to have problems falling asleep; the observation from a study that exercise of sufficient intensity may help men to fall asleep and to sleep for a longer duration [32], could be useful for countering sleep deprivation and improved life quality.

If sleepiness, inadequate off-duty “recovery sleep”, and unhealthy life style are factors that appear interallied, and their association with quality of life is consistent, the poor quality of life in young doctors unmarried seems a particular factor. We hypothesis, this factor still related to unhealthy lifestyle. If married physicians have more responsibilities with their families, who need more presence in the family home. The young unmarried physicians may spend more time outside their homes with evening activities with friends, this magnified the effects of inadequate sleep in medical setting by additional inadequate “recovery sleep”.

Our study has several limitations. Perhaps the most important is that it was conducted in a single residency program in 1 institution, which limits the ability to generalize the results. Second, the self-reporting of sleep/wake habits relies on the training physicians’ subjective accounts, which raises the possibility that the training physicians may not have accurately reported their sleep habits. Moreover, Wolfson and colleagues have demonstrated the validity of self-reported survey estimates of sleep patterns in adolescents [33]. Third, the group of participating physicians had a mean age of 26 ans. Younger physicians are more likely to accept a rotation schedule in Morocco. Results can thus not be generalized to older physicians.

In conclusion, this study showed that two third (66.7%) of training physicians had suffered from sleepiness. There was an association between poor quality of life and severe sleepiness in unmarried physicians, sleeping less than 6 hours in shift-off day, and doing no physical activity. Because healthcare delivery in hospitals must operate 24 hours a day, 7 days a week, Managing shift work (shift work strategies, duty hour limitations with recovery. Work-hour limits) have fueled a vigorous discussion of the optimal balance of continuity of patient care, physician well-being, and trainee education. Thus, health care professionals need to develop a sense of both their personal vulnerability to fatigue and the approaches that work best for them and their lifestyle. Then, the question who arises: we should reduce the hours of work or rather improve the working conditions of doctors? Only a handful of studies have specifically addressed the issue of countermeasure strategies in healthcare professionals, we proposed:

Napping which provide temporary relief from the effects of sleep loss and fatigue, the optimal duration of brief naps is 15 to 20 min so individuals napping for this short duration are likely to avoid going into deep sleep, and thus the effects of sleep inertia upon awaking are minimized, napping strategies may include “prophylactic” naps before work, or splitting sleep into two 4-hour periods.

Sleep hygiene, good sleep habits to improve the strength of the circadian rhythm, relaxing pre sleep rituals, and a comfortable sleep environment will lead to improved sleep quality and quantity:

Strengthen the team of doctors on call;

Providing measures of improvement of the lifestyle within the hospital is required.

## Competing interests

The authors declare that they have no competing interests.

## Authors’ contributions

JB conceived the study, performed the statistical analysis, and drafted the manuscript. OB participated in the design of the study, in acquisition of data, and in manuscript drafting. NM, KA, and TD participated in the coordination of data; AAZ participated in study design. RA conceived the study, participated in the design of the study, performed the statistical analysis and interpretation of data, and gave the final approval of the manuscript. All coauthors read and approved the final manuscript. There is no financial interest to report.
